# Structure-Function Relationships of the *Mycobacterium tuberculosis* Transcription Factor WhiB1

**DOI:** 10.1371/journal.pone.0040407

**Published:** 2012-07-05

**Authors:** Laura J. Smith, Melanie R. Stapleton, Roger S. Buxton, Jeffrey Green

**Affiliations:** 1 Department of Molecular Biology and Biotechnology, University of Sheffield, Sheffield, United Kingdom; 2 Division of Mycobacterial Research, Medical Research Council National Institute for Medical Research, Mill Hill, London, United Kingdom; Saint Louis University, United States of America

## Abstract

**Background:**

Members of the WhiB-like (Wbl) protein family possess iron-sulfur clusters and are implicated in the regulation of developmental processes in Actinomycetes. *Mycobacterium tuberculosis* possesses seven Wbl proteins. The [4Fe-4S] cluster of *M. tuberculosis* WhiB1 is relatively insensitive to O_2_ but very sensitive to nitric oxide (NO). Nitric oxide nitrosylates the WhiB1 iron-sulfur cluster and promotes DNA-binding; the apo-forms of WhiB1 also bind DNA. However, the molecular requirements for iron-sulfur cluster acquisition and for DNA-binding by WhiB1 are poorly characterized.

**Methods and Findings:**

WhiB1 variants were created by site-directed mutagenesis and the abilities of the corresponding proteins to acquire an iron-sulfur cluster and/or bind to *whiB1* promoter DNA were assessed. All four Cys residues (Cys9, 37, 40, and 46) in the N-terminal region of WhiB1 were required for incorporation of a [4Fe-4S] cluster, whereas a possible alternative cluster ligand Asp13 (by analogy with *M. smegmatis* WhiB2) was not. The C-terminal region of WhiB1 is predicted to house the DNA-binding domain of the protein consisting of a predicted β-turn (^58^GVWGG^62^) followed by two amino acid motifs (^72^KRRN^75^ and ^78^TKAR^81^) that are conserved in WhiB1 proteins. Gly residues (Gly58, 61 and 62) in the β-turn and positively-charged residues (Lys72, Arg73, Arg74, Lys79 and Arg81) in the downstream conserved regions were required for binding of WhiB1 DNA.

**Conclusions:**

Site-directed mutagenesis of *M. tuberculosis whiB1* and characterization of the corresponding proteins has been used to explore structure-function relationships of the NO-responsive transcription factor WhiB1. This showed that all four conserved Cys residues in the N-terminal region are required for incorporation of iron-sulfur clusters but not for DNA-binding. Analysis of variants with amino acid substitutions in the C-terminal region revealed the crucial roles played by a predicted β-turn and two conserved positively-charged motifs in facilitating DNA-binding, but not iron-sulfur cluster acquisition, by WhiB1.

## Introduction

*M. tuberculosis* is the causative agent of tuberculosis (TB) and is a major worldwide healthcare problem [1]. As is the case for many bacterial pathogens, the ability of *M. tuberculosis* to cause disease requires efficient and effective gene regulation to survive within the hostile environment of the host. Nitric oxide (NO) production by lung macrophages (the preferred niche for *M. tuberculosis*) is a major component of the host defenses and *M. tuberculosis* has evolved mechanisms to sense NO and reprogram gene expression to counteract the deleterious effects of reactive nitrogen species [2]–[4]. It has recently been shown that the *M. tuberculosis* WhiB1 protein is an essential iron-sulfur protein that upon exposure to NO switches from a non-DNA-binding holo-form to a DNA-binding nitrosylated transcription factor; the apo-forms of WhiB1 also bind DNA [5]–[7]. The ability to sense and respond to NO is an important adaptive feature of many bacterial pathogens because it is a crucial component of the host innate immune response [8]. The preferred niche of *M. tuberculosis* is the lung macrophage where it is exposed to NO generated by inducible NO synthase (iNOS). Upon exposure to NO *M. tuberculosis* initiates a complex reprogramming of gene expression to counteract the deleterious effects of reactive nitrogen species [2], [3]. Furthermore, it has been suggested that NO is a key signal in promoting transition from active *M. tuberculosis* growth to the dormant state, thereby contributing to the burden of latent TB infections [3], [9]. Thus, there is much interest in the mechanisms of NO-perception and subsequent transduction of this signal into altered patterns of gene expression.

WhiB1 is a member of the WhiB-like (Wbl) protein family that is associated with the regulation of developmental processes in Actinomycetes [10]. Like other members of the Wbl family, WhiB1 possesses four conserved Cys residues (Cys9, 37, 40 and 46) in its N-terminal region that are proposed to act as the ligands for a [4Fe-4S] cluster [5], [6]. The WhiB1 iron-sulfur cluster is relatively stable in the presence of O_2_ but is extremely reactive with NO [5], [6]. Exposure of WhiB1 to NO results in cluster nitrosylation and conversion of the protein from a non-DNA-binding form to one capable of interacting with target DNA (including the *whiB1* promoter itself and the *groEL2* promoter) to repress transcription [5], [7]. In addition to DNA-binding by nitrosylated WhiB1, the reduced (thiol) and oxidized (disulfide) forms of apo-WhiB1 also bind DNA [5]. Hence, WhiB1-mediated transcription regulation is dependent upon the presence or absence and state (nitrosylated or non-nitrosylated) of the [4Fe-4S] cluster, as well as the redox state of apo-WhiB1 [5], [7]. Therefore, it is becoming clear that nitrosylation and/or disassembly of the WhiB1 iron-sulfur cluster triggers DNA-binding, but how Wbl proteins bind DNA is poorly characterized.

The C-terminal region of WhiB1 is predicted to house the DNA-binding domain of the protein. It contains a motif (GVWGG followed by a region of positively charged amino acids) that has been suggested to form the β-turn and DNA-recognition helix of an unconventional helix-turn-helix motif. However, before the present work there was no experimental evidence that these features were associated with the DNA-binding activity of WhiB1, although this region has been implicated in DNA-binding by WhiBTM4, a Wbl protein encoded by mycobacteriophage TM4 [11]. Thus, in WhiBTM4 the amino acid substitutions L55Q, V56D, V58E (in the β-turn) and R67C (in the putative DNA-recognition helix) significantly impaired DNA-binding [11].

Based on the evidence described above, a picture is emerging of WhiB1 as an essential transcription factor involved in reprogramming *M. tuberculosis* gene expression in response to NO generated by infected host macrophages. However, little is known of the fundamental mechanisms that underpin the ability of WhiB1 to acquire an iron-sulfur cluster and bind DNA. Here site-directed mutagenesis has been used to create WhiB1 variants. Analysis of these variants showed that all four conserved N-terminal Cys residues are required for iron-sulfur cluster acquisition and that amino acid residues in the predicted β-turn (Gly58, 61 and 62) and in two conserved motifs located downstream of the β-turn (Lys72, Arg73, Arg74, Lys79 and Arg81) are required for DNA-binding.

## Results

### All four WhiB1 Cys residues are necessary for acquisition of a [4Fe-4S] cluster

**Figure 1:**
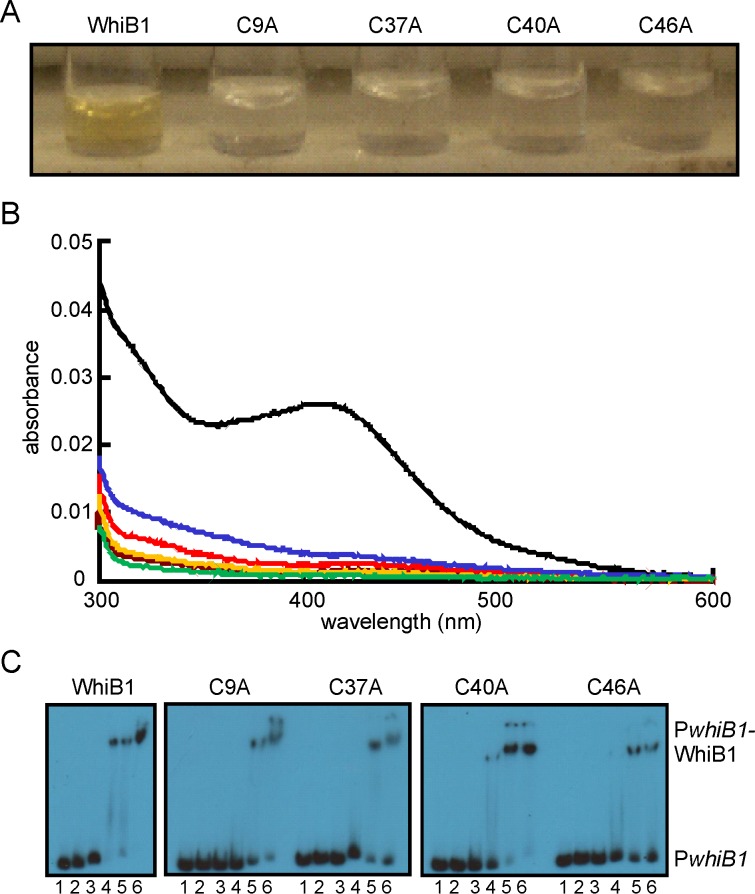
All four Cys residues of WhiB1 are required for iron-sulfur cluster acquisition. (A) Photograph depicting anaerobic reconstitution of WhiB1 and variants with the indicated single Cys substitutions after removal of unincorporated components by chromatography on heparin Sepharose. (B) UV-visible spectra of WhiB1 and variants after anaerobic reconstitution of iron-sulfur clusters. The black line shows the spectrum of wild-type WhiB1 (4.5 µM); the blue line, WhiB1-C9A (2.5 µM); the red line, WhiB1-C37A (3.7 µM); the brown line, WhiB1-C40A (3.6 µM); the orange line, WhiB1-C46A (3.6 µM). The green line shows the spectrum of apo-WhiB1 (2.3 µM). The buffer was 25 mM Tris-HCl pH 7.4 containing 0.5 M NaCl and 10% glycerol. (C) All four WhiB1 Cys variants bind *whiB1* promoter DNA (P*whiB1*). Radiolabeled P*whiB1* DNA was incubated with increasing concentrations of the indicated WhiB1 proteins before separation of protein-DNA complexes in electrophoretic mobility shift assays. Lanes 1, no protein; lanes 2–6 contain, 1, 2, 5, 10 and 15 μM WhiB1, respectively. The locations of P*whiB1* and P*whiB1*-WhiB1 complexes are indicated.

**Figure 2:**
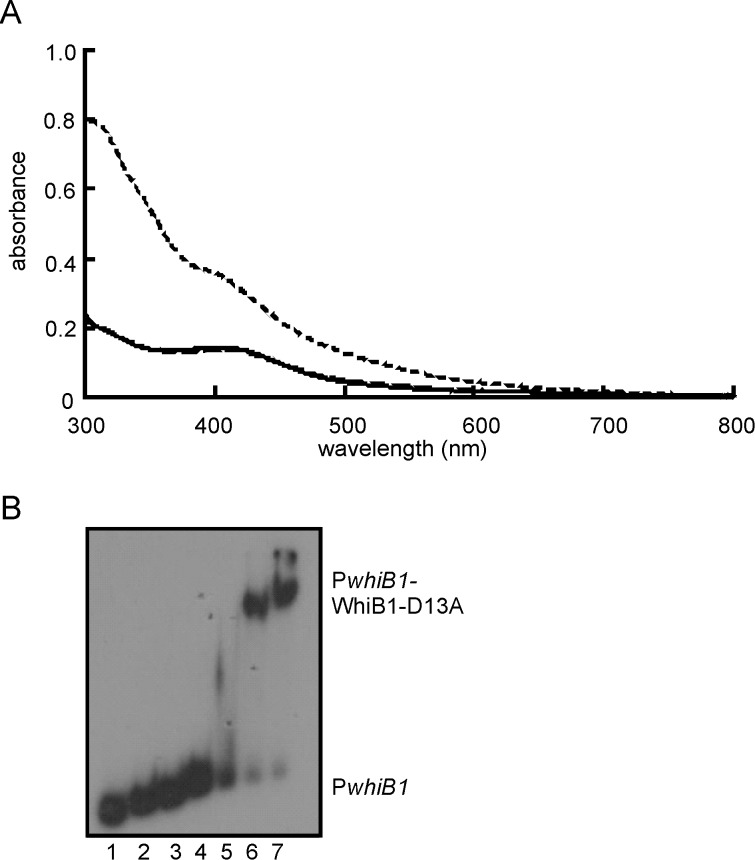
The WhiB1-D13A variant has properties similar to the wild-type protein. (A) Spectroscopic properties and reactivity of the WhiB1-D13A iron sulfur cluster. The UV-visible spectra of WhiB1-D13A (18.8 μM): after reconstitution (solid line); after 60 min exposure to 110 μM O_2_ (long-dashed line; mostly superimposed on the solid line); and after 5 min exposure to a 20-fold molar excess of NO (short-dashed line). The buffer was 25 mM Tris-HCl pH 7.4 containing 0.5 M NaCl, 1 mM DTT and 10% glycerol. (B) Apo-WhiB1-D13A binds P*whiB1*. Electrophoretic mobility shift assays were as follows: lanes 1, no protein; lanes 2–7, 0.5, 1, 2, 4, 8 and 16 μM WhiB1-D13A, respectively. The locations of P*whiB1* and P*whiB1*-WhiB1-D13A complexes are indicated.

Wbl proteins are characterized by the presence of four conserved Cys residues that are thought to act as the anchors for iron-sulfur clusters (either [4Fe-4S] or [2Fe-2S]) [10]. However, substitution of one of the Cys residues (Cys67) in *M. smegmatis* WhiB2 did not affect *in vivo* activity but replacement of Asp71 did, suggesting that Cys67 was not required for WhiB2 function, whereas Asp71 was; the equivalents of Cys67 (Cys9) and Asp71 (Asp13) are conserved in WhiB1 [12]. Therefore, to identify the amino acid residues required for incorporation of an iron-sulfur cluster into WhiB1, the four Cys residues (Cys9, Cys37, Cys40 and Cys46) and Asp13 were singly replaced by Ala and the ability of the corresponding proteins to acquire an iron-sulfur cluster was assessed by anaerobic reconstitution [5]. The wild-type protein was straw-brown colored (Figure 1A) and displayed an absorbance spectrum with a shoulder at 420 nm (Figure 1B), typical of proteins with a [4Fe-4S] cluster. By contrast, the Cys variants, either as prepared or after anaerobic reconstitution lacked color (Figure 1A) and had absorbance spectra that resembled that of the apo-WhiB1 protein (Figure 1B). The A_420_∶A_280_ ratios were: 0.35, wild type; 0.06, C9A; 0.06, C37A; 0.04, C40A; 0.09, C46A; and 0.02, apo-WhiB1. In contrast to WhiB2, replacement of Asp13 by Ala did not affect the capacity of WhiB1 to acquire an iron-sulfur cluster; the WhiB1-D13A variant had an absorption spectrum identical to that of the wild-type protein, A_420_∶A_280_ ratio 0.36 (Figure 2A). Accordingly, the WhiB1-D13A variant iron-sulfur cluster (18.8 μM) was stable in the presence of O_2_ (5-fold molar excess) but reacted rapidly (reaction was almost complete within 1 min at 25°C) with NO (20-fold molar excess) producing an absorption spectrum with enhanced absorbance in the 360 nm region, typical of a protein with a nitrosylated iron-sulfur cluster (Figure 2A). Thus, it was concluded that all four Cys residues (but not Asp13) of WhiB1 were required for iron-sulfur cluster acquisition.

The apo-forms of WhiB1 are able to bind to *whiB1* promoter DNA (P*whiB1*) whereas the holo- ([4Fe-4S]) form cannot [5]. Therefore, it was expected that the WhiB1 Cys variants, which all failed to acquire an iron-sulfur cluster, would retain the ability to bind DNA if they were correctly folded. This proved to be the case; although there appeared to be minor differences in binding affinity, overall the WhiB1 Cys variants bound P*whiB1* similarly to the wild-type protein (Figure 1C). Furthermore, the DNA-binding properties of the apo-form of WhiB1-D13A were also similar to those of the wild-type protein (Figure 2B).

### Amino acid residues in a predicted β-turn and in two conserved motifs in the C-terminal region of WhiB1 are required for DNA-binding

**Figure 3:**
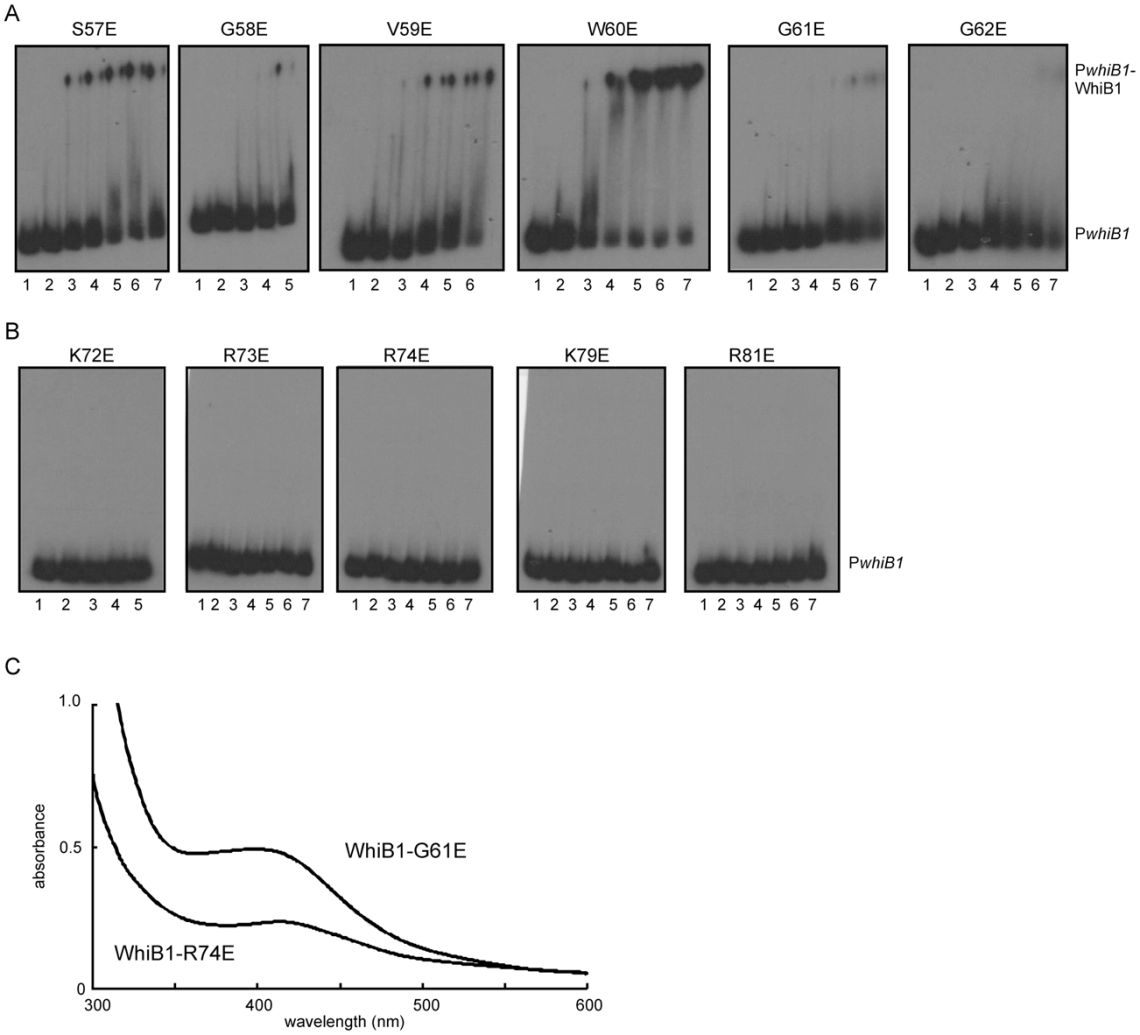
DNA-binding and iron-sulfur cluster acquisition by WhiB1 variants with amino acid substitutions in the C-terminal region. WhiB1 proteins with the indicated amino acid substitutions were incubated with radiolabeled P*whiB1* DNA and complexes were separated by electrophoresis. (A) Amino acid substitutions in the putative β-turn of WhiB1. (B) Amino acid substitutions in two conserved amino acid motifs located downstream of the predicted β-turn of WhiB1. Lanes 1, no protein; lanes 2–7, 2.5, 5, 7.5, 10, 12.5 and 15 μM of the indicated WhiB1 protein, respectively. The locations of P*whiB1* and P*whiB1*-WhiB1 complexes are indicated. (C) Representative UV-visible spectra of holo- WhiB1-G61E (26 μM; A_420_∶A_280_ ratio 0.19) and WhiB1-R74E (13 μM; A_420_∶A_280_ ratio 0.16).

Previous work has shown that apo- and nitrosylated-WhiB1 binds specifically to P*whiB1* DNA [5]. The C-terminal region of WhiB1 is predicted to house the DNA-binding domain. Sequence analysis and secondary structure predictions suggest the presence of a β-turn (^58^GVWGG^62^) followed by a predicted helical region (^65^EDERRALKRRNA^76^). Replacement of Gly residues 58, 61 or 62 in the β-turn by Glu inhibited DNA-binding by WhiB1, whereas replacement of Ser57, Val59, or Trp60 by Glu did not significantly impair the ability of WhiB1 to bind P*whiB1* DNA in electrophoretic mobility shift assays (Figure 3A). UV-visible spectra of the WhiB1 variants with impaired DNA-binding showed that they were able to acquire an iron-sulfur cluster, suggesting that the amino acid replacements had not severely disrupted the overall fold of these proteins Figure 3C). These data indicate that the disruption of the structural integrity imposed by the Gly residues of the predicted β-turn severely inhibits DNA-binding.

**Figure 4:**
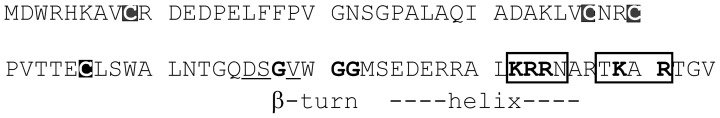
Locations of amino acids required for iron-sulfur cluster acquisition and DNA-binding by WhiB1. The locations of: the four conserved Cys residues that are required for iron-sulfur cluster acquisition (white text on black background); the predicted β-turn and helix in the C-terminal region; the WhiB1 conserved motifs downstream of the predicted β-turn (boxed); and amino acids that impair DNA-binding in WhiB1 (bold) or WhiBTM4 (underlined) are shown.

Amino acid sequence alignment of 29 Wbl proteins using ClustalW [13] identified two regions (^72^KRRN^75^ and ^78^TKAR^81^) located downstream of the predicted β-turn that are conserved in WhiB1 proteins (Figure 4 and Figure S1). Single replacements of positively-charged residues (Lys72, Arg73, Arg74, Lys79 and Arg81) in these regions by negatively-charged Glu abolished DNA-binding (Figure 3B). However, these WhiB1 variants were capable of incorporating iron-sulfur clusters (Figure 3C) and therefore the inability to bind DNA was judged not to be due to a general disruption of the protein fold.

## Discussion

Recently, mycobacterial Wbl proteins have been recognized as transcription factors with functions that can be modulated by NO; however relatively little is known of the structure-function relationships of this family of proteins [5]–[7], [11], [14]. Here it is shown that all four Cys residues of WhiB1 are essential for iron-sulfur cluster acquisition.

The most common ligands for iron-sulfur clusters in proteins are four Cys residues. Accordingly, simultaneous replacement of all four conserved Cys residues of WhiB3, which is involved in maintaining redox homeostasis and surviving nutrient starvation, abolished the ability to bind an iron-sulfur cluster [14]. Similarly, the *in vivo* function of the *Streptomyces coelicolor* Wbl protein WhiD in late stage sporulation required all four conserved Cys residues [15]. Surprisingly, only three of the four conserved Cys residues were required for *M. smegmatis* WhmD function in cell septation and division with a conserved Asp residue being implicated as the fourth cluster ligand [12]. Mycobacteriophage WhiBTM4 interferes with cell septation and variants with C36S or C45S substitutions retained some ability to bind iron-sulfur clusters, albeit ones with increased O_2_ sensitivity [11]. Here it is shown that the requirements for iron-sulfur cluster acquisition by WhiB1 resemble those of *S. coelicolor* WhiD, i.e. all four conserved Cys residues (but not the conserved Asp residue) are necessary for cluster incorporation (Figures 1 and 2) [15]. The WhiB1 iron-sulfur cluster is unusually stable in the presence of O_2_ but very sensitive to NO [5], [6]; these properties were not affected by replacement of the conserved Asp residue (Asp13), which is a likely cluster ligand and required for the function of WhmD [12]. In contrast to WhiB1, WhiBTM4 and WhiB3 exhibited significant cluster degradation in the presence of air [11], [14]. The specific properties of Wbl iron-sulfur clusters will be determined by the particular protein environment in which they are housed, therefore from a structure-function viewpoint there are likely to be amino acid side-chains in the vicinity of the iron-sulfur of WhiB1 that contribute to its insensitivity to O_2_ allowing it to act as an aerobic NO sensor. Further targeted and/or random mutagenesis studies are necessary to identify such residues.

To the authors' knowledge there has only been one other attempt to define the molecular requirements for DNA-binding by Wbl proteins. A screen for loss of function mutants of the mycobacteriophage TM4 Wbl protein WhiBTM4 and subsequent analysis by electrophoretic mobility shift assays indicated that amino acid substitutions in the predicted C-terminal β-turn (L55Q, V56D, and V58E) inhibited DNA-binding [11]. For WhiB1 replacement of the conserved Gly residues (Gly58, 61 and 62) of the β-turn by Glu inhibited DNA-binding (Figures 3A and 4). Thus, it appears that the structural integrity of the predicted β-turn is necessary for DNA-binding by WhiB1 and WhiBTM4. A fourth WhiBTM4 mutation that resulted in impaired DNA-binding was the replacement of Arg67 in the predicted helix located C-terminal of the β-turn by Cys [11]. Therefore, two conserved positively-charged motifs in the C-terminal regions of WhiB1 proteins were targeted for mutagenesis. The resulting singly-substituted WhiB1 variants all lacked the ability to bind DNA (Figure 3B), whilst retaining the capacity to acquire iron-sulfur clusters (Figure 3C). Thus, it appears that several positively-charged amino acid residues in the C-terminal region, as well as the β-turn, are required for DNA-binding by WhiB1 and WhiBTM4, and the introduction of negatively-charged residues into this region of WhiB1 severely inhibits DNA-binding.

Iron-sulfur cluster-mediated inhibition of DNA-binding is presumably achieved by conformational changes in the C-terminal region of WhiB1 that are imposed by the [4Fe-4S] cluster. That the state of the WhiB1 iron-sulfur cluster has the potential to induce conformational changes in the protein was suggested by its elution profile during purification by Ni-affinity chromatography in which the [4Fe-4S] form eluted before the apo-form, which in turn eluted before a [2Fe-2S] form; the cluster state of the protein fractions was assigned by UV-visible spectroscopy. This fractionation of WhiB1 forms is interpreted as a reflection of different conformations associated with the different states of the iron-sulfur cluster, each with the potential to modulate the DNA-binding properties of the protein. Thus, the experiments to explore WhiB1 structure-function relationships have shown that at least three C-terminal amino acid motifs are necessary for DNA-binding and that interaction with DNA is inhibited by the presence of a [4Fe-4S] cluster ligated by Cys9, Cys37, Cys40 and Cys46. Hence, this work provides the foundation for the development of a comprehensive structure-function map of DNA-binding Wbl proteins.

## Materials and Methods

### Isolation of proteins and creation of site-directed mutants

The WhiB1 proteins were isolated as hexa-His-tagged proteins by affinity chromatography on Hi-Trap Chelating columns (1 ml; GE Healthcare) as described previously [5]. Oligonucleotides (Eurofins MWG Operon) were designed to alter the desired codon in the *whiB1* gene of the expression plasmid pGS2164 using the Quikchange II SDM Kit (Stratagene). The authenticities of the resulting plasmids were confirmed by DNA sequencing (Source Bioscience). Reconstitution reactions and removal of unincorporated reagents by heparin chromatography were as previously described [5], [6].

### Electrophoretic mobility shift assays

The *whiB1* promoter region (P*whiB1*) was amplified from the pGS2060 plasmid and consisted of a DNA fragment extending from −177 to +108 relative to the transcript start [5]. The resulting DNA was digested with XbaI and end labelled using 0.37 MBq of [α-^32^P]dCTP and Klenow enzyme (10 units) for 60 min at 25°C. Unincorporated radionucleotides were removed using a QIAquick PCR clean-up kit (Qiagen). Radiolabeled DNA (∼3 ng) was incubated with 0–16 μM of the indicated His-WhiB1 variants in the presence of 100 mM NaCl, 40 mM Tris (pH 8), 10 mM MgCl_2_, 1 mM EDTA, 250 μg ml^−1^ BSA, 1 mM DTT, and calf thymus DNA (300-fold excess). Reactions were incubated on ice for 10 min before the resulting complexes were separated on 6% polyacrylamide gels buffered with 45 mM Tris-Borate containing 1 mM EDTA.

### UV-visible spectroscopy

Scanning spectroscopy was carried out using a Cary 50 Bio UV-Vis spectrophotometer using Hellma 10 mm cuvettes fitted with a screw top lid to maintain anaerobic conditions.

## Supporting Information

Figure S1 **Amino acid alignment of 29 WhiB proteins.** The alignment was made using ClustalW (Thompson et al., 1994, *Nucl. Acids Res*. 22: 4673–4680.). In the protein list on the right WhiB1 proteins are highlighted in red (*M. tuberculosis* WhiB1 is Rv3219). Protein accession numbers in Genbank are provided on page 1. Cysteine residues (yellow); for the WhiB1 proteins the predicted β-turn (green); and two conserved motifs (blue) in the C-terminal region are indicated. The numbers on the left are the positions of the first amino acid in that row in the primary structure of the indicated protein. A boldface **M** or **L** indicates possible alternative N-termini.
(RTF)
