# Supplementary material for: Structure-Function Relationships of the Mycobacterium tuberculosis Transcription Factor WhiB1
Source: PLoS One. 2012 Jul 5;7(7):e40407. doi: 10.1371/journal.pone.0040407 (PMC3390391; doi:10.1371/journal.pone.0040407)
Supplement: Figure S1 — Amino acid alignment of 29 WhiB proteins. The alignment was made using ClustalW (Thompson et al., 1994, Nucl. Acids Res. 22: 4673–4680.). In the protein list on the right WhiB1 proteins are highlighted in red (M. tuberculosis WhiB1 is Rv3219). Protein accession numbers in Genbank are provided on page 1. Cysteine residues (yellow); for the WhiB1 proteins the predicted β-turn (green); and two conserved motifs (blue) in the C-terminal region are indicated. The numbers on the left are the positions of the first amino acid in that row in the primary structure of the indicated protein. A boldface M or L indicates possible alternative N-termini. (RTF) [file pone.0040407.s001.rtf]

   1  - - - - - - - - - - - - - - - - - - - - - - - - - - - - - -  WHIBTM4  NP_569784.1
   1  - - - - - - - - - - - - - - - - - - - - - - - - - - - - - -  CGLwhiB1 
   1  - - - - - - - - - - - - - - - - - - - - - - - - - - - - - -  SCOELWB ABU39981.1
   1  - - - - - - - - - - - - - - - - - - - - - - - - - - - - - -  MB3706c NP_857345.1 
   1  - - - - - - - - - - - - - - - - - - - - - - - - - - - - - -  MB3221c  NP_856866.1
   1  - - - - - - - - - - - - - - - - - - - - - - - - - - - - - -  MB3245  YP_005172698.1
   1  - - - - - - - - - - - - - - - - - - - - - - - - - - - - - -  MB3288c  NP_856933.1
   1  - - - - - - - - - - - - - - - - - - - - - - - - - - - - - -  MB3450 NP_857090.1
   1  - - - - - - - - - - - - - - - - - - - - - - - - - - - - - -  ML2307c NP_302502.1
   1  - - - - - - - - - - - - - - - - - - - - - - - - - - - - - -  ML0382c NP_301374.1
   1  - - - - - - - - - - - - - - - - - - - - - - - - - - - - - -  ML0639  NP_301528.1
   1  - - - - - - - - - - - - - - - - - - - - - - - - - - - - - -  ML0760  NP_301589.1
   1  - - - - - - - - - - - - - - - - - - - - - - - - - - - - - -  ML0804c  NP_301614.1
   1  - - - - - - - - - - - - - - - - - - - - - - - - - - - - - -  MAPwhib1 ZP_09973330.1
   1  - - - - - - - - - - - - - - - - - - - - - - - - - - - - - -  MAviumwhiB1NP_962254.1
   1  - - - - - - - - - - - - - - - - - - - - - - - - - - - - - -  MAvium290  YP_883290.1
   1  - - - - - - - - - - - - - - - - - - - - - - - - - - - - - -  MAvium364  YP_883364.1
   1  - - - - - - - - - - - - - - - - - - - - - - - - - - - - - -  MAvium496 YP_883496.1
   1  - - - - - - - - - - - - - - - - - - - - - - - - - - - - - -  MAvium729 YP_879729.1
   1  - - - - - - - - - - - - - - - - - - - - - - - - - - - - - -  MS6199  YP_890419.1
   1  - - - - - - - - - - - - - - - - - - - - - - - - - - - - - -  MS1597  YP_885976.1
   1  - - - - - - - - - - - - - - - - - - - - - - - - - - - - - -  MS1831  YP_886201.1
   1  - - - - - - - - - - - - - - - - - - - - - - - - - - - - - -  MS1919  YP_886285.1	
   1  L A G R R C H C A A A Q A S S H R R R H R D K R V R G S E P  MS1953  YP_886319.1
   1  - - - - - - - - - - - - - - - - - - - - - - - - - - - - - -  Rv3681c CAA18003.2
   1  - - - - - - - - - - - - - - - - - - - - - - - - - - - - - -  Rv3197A CAE55564.1
   1  - - - - - - - - - - - - - - - - - - - - - - - - - - - - - -  Rv3219  CAB08318.1
   1  - - - - - - - - - - - - - - - - - - - - - - - - - - - - - -  Rv3260c CAA17074.1
   1  - - - - - - - - - - - - - - - - - - - - - - - - - - - - - -  Rv3416  CAB01007.1


      
   1  - - - - - - - - - - - - - - - - - - - - - - - - - - - - - -  WHIBTM4
   1  - - - - - - - - - - - - - - - - - - - - - - - - - - - - - -  CGLwhiB1
   1  - - - - - - - - - - - - - - - - - - - - - - - - - - - - - -  SCOELWB!
   1  - - - - - - - - - - - - - - - - - - - - - - - - - - - - - -  MB3706c
   1  - - - - - - - - - - - - - - - - - - - - - - - - - - - - - -  MB3221c
   1  - - - - - - - - - - - - - - - - - - - - - - - - - - - - - -  MB3245
   1  - - - - - - - - - - - - - - - - - - - - - - - - - - - - - -  MB3288c
   1  - - - - - - - - - - - - - - - - - - - - - - - - - - - - - -  MB3450
   1  - - - - - - - - - - - - - - - - - - - - - - - - - - - - - -  ML2307c
   1  - - - - - - - - - - - - - - - - - - - - - - - - - - - - - -  ML0382c
   1  - - - - - - - - - - - - - - - - - - - - - - - - - - - - - -  ML0639
   1  - - - - - - - - - - - - - - - - - - - - - - - - - - - - - -  ML0760
   1  - - - - - - - - - - - - - - - - - - - - - - - - - - - - - -  ML0804c
   1  - - - - - - - - - - - - - - - - - - - - - - - - - - - - - -  MAPwhib1
   1  - - - - - - - - - - - - - - - - - - - - - - - - - - - - - -  MAviumwhiB1
   1  - - - - - - - - - - - - - - - - - - - - - - - - - - - - - -  MAvium290
   1  - - - - - - - - - - - - - - - - - - - - - - - - - - - - - -  MAvium364
   1  - - - - - - - - - - - - - - - - - - - - - - - - - - - - - -  MAvium496
   1  - - - - - - - - - - - - - - - - - - - - - - - - - - - - - -  MAvium729
   1  - - - - - - - - - - - - - - - - - - - - - - - - - - - - - -  MS6199
   1  - - - - - - - - - - - - - - - - - - - - - - - - - - - - - -  MS1597
   1  - - - - - - - - - - - - - M S Y E S G D F D R V V R F D N R  MS1831
   1  - - - - - - - - - - - - - - - - - - - - - - - - - - - - - -  MS1919
  31  L L G R G F T P A E K A T D P Q S P G S V A I F V G P P E K  MS1953
   1  - - - - - - - - - - - - - - - - - - - - - - - - - - - - - -  Rv3681c
   1  - - - - - - - - - - M A T D P K S Q D P W P N F R D I C P P  Rv3197A
   1  - - - - - - - - - - - - - - - - - - - - - - - - - - - - - -  Rv3219
   1  - - - - - - - - - - - - - - - - - - - - - - - - - - - - - -  Rv3260c
   1  - - - - - - - - - - - - - - - - - - - - - - - - - - - - - -  Rv3416


   1  - - - - - - - - - - - - - - - - - - - - - - - - - - - - - -  WHIBTM4
   1  - - - - - - - - - - - - - - - - - - - - - - - - - - - - - -  CGLwhiB1
   1  - - - - - - - - - - - - - - - - - - - - - - - - - - - - - -  SCOELWB!
   1  - - - - - - - - - - - - - - V S G T R P A A R R T N - L T A  MB3706c
   1  - - - - - - - - - - - - - - - - - - - - - - - - - - - V S V  MB3221c
   1  - - - - - - - - - - - - - - - - - - - - - - - - - - - - - -  MB3245
   1  - - - - - - - - - - - - - - - - - - - - - - - - - - - L V P  MB3288c
   1  - - - - - - - - - - - - - - - - - - - - - - - - - - - - M P  MB3450
   1  - - - - - - - - - - - - - - V A K T R P A A R R T N - L S A  ML2307c
   1  - - - - - - - - - - - - - - - - - - - - - - - - - - - - M P  ML0382c
   1  - - - - - - - - - - - - - - - - - - - - - - - - - - - M L T  ML0639
   1  - - - - - - - - - - - - - - - - - - - - - - - - - - - V V P  ML0760
   1  - - - - - - - - - - - - - - - - - - - - - - - - - - - - - -  ML0804c
   1  - - - - - - - - - - - - - - - - - - - - - - - - - - - - - -  MAPwhib1
   1  - - - - - - - - - - - - - - - - - - - - - - - - - - - - - -  MAviumwhiB1
   1  - - - - - - - - - - - - - - - - - - - - - - - - - - - M S A  MAvium290
   1  - - - - M A G T P H T P I D S A P A R P V - R P H L T V V P  MAvium364
   1  - - - - - - - - - - - - - - - - - - - - - - - - - - - - - -  MAvium496
   1  - - - - - - - - - - - - - - - - - - - - - - - - - - - - - -  MAvium729
   1  - - - - - - - - - M K G S L V S A T H T V A R K A S T L S T  MS6199
   1  - - - - - - - - - - - - - - - - - - - - - - - - - - - - M P  MS1597
  18  L L G S V S H A P H I D T G S T P T G A A G R P Q L S L V P  MS1831
   1  - - - - - - - - - - - - - - - - - - - - - - - - - - - - - -  MS1919
  61  S G R R I H Q L R Q I T D K F D Q M Q G E K H M S I A M T A  MS1953
   1  - - - - - - - - - - - - - - V S G T R P A A R R T N - L T A  Rv3681c
  21  Q H P W S G S L - - - - - S K N R H Q T R K Q V K Q D V S V  Rv3197A
   1  - - - - - - - - - - - - - - - - - - - - - - - - - - - - - -  Rv3219
   1  - - - - - - - - - - - - - - - - - - - - - - - - - - - L V P  Rv3260c
   1  - - - - - - - - - - - - - - - - - - - - - - - - - - - - M P  Rv3416


   1  - - - - - - - - - - - - M H M H M G G D P S A I C A Q T D P  WHIBTM4
   1  - - - - - - - - - - - - - - - - M D W R H - - A C R - - D -  CGLwhiB1
   1  - - - - - - - - - - - - - - - - - - W Q E R A L C A Q T D P  SCOELWB!
  16  A Q N - - - V V R S V D A E E R I A W V S K A L C R T T D P  MB3706c
   4  L T - - - - - - - - V P R Q T P R Q R L P V L P C H V G D P  MB3221c
   1  - - - - - - - - - - - - - - - - M D W R H K A V C R D E D P  MB3245
   4  E A P A P F E - E P L P P E A T D Q W Q D R A L C A Q T D P  MB3288c
   3  Q P - - - - E Q L P G P N A D I W N W Q L Q G L C R G M D S  MB3450
  16  A K S - - - P L H S V D A E E R I A W V S K A L C R A T D P  ML2307c
   3  Q P - - - - K Q L P G P N A T I W N W Q L Q G L C R G V D S  ML0382c
   4  L T - - - - - - - - I P K Q T - - - - L P G L P C H A D T S  ML0639
   4  K A L V A F E V E S E P E - S S D Q W Q D R A L C A Q T D P  ML0760
   1  - - - - - - - - - - - - - - - - M D W R H K A V C R D E D P  ML0804c  
   1  - - - - - - - - - - - - - - - - M D W R H K A V C R D E D P  MAPwhib1
   1  - - - - - - - - - - - - - - - - M D W R H K A V C R D E D P  MAviumwhiB1
   4  P T - - - - - - - - V P R - - - - - - - Q A L P C H V G D P  MAvium290
  26  D A P V A F E P E P L P A P V A D Q W Q D R A L C A Q T D P  MAvium364
   1  - - - - - - - - - - - - - - - - - - - - M H G L C R G V D S  MAvium496
   1  - - - - - - - - - - - - - - - - - - - - - - - - - - - - - -  MAvium729
  22  T T N - - - T A Q H R D S E A R I A W V S Q A R C R Q A D P  MS6199
   3  Q P - - - - Q Q L P G P N A D I W D W Q M R G L C R G V D S  MS1597
  48  D S - - - F D V A P E A E - - E D Q W Q E R A L C A Q T D P  MS1831
   1  - - - - - - - - - - - - - - - - M D W R H K A V C R D E D P  MS1919
  91  P T T - - - - - G V A P M T C - E T R L P A V P C H V G D P  MS1953
  16  A Q N - - - V V R S V D A E E R I A W V S K A L C R T T D P  Rv3681c
  46  L T - - - - - - - - V P R Q T P R Q R L P V L P C H V G D P  Rv3197A
   1  - - - - - - - - - - - - - - - - M D W R H K A V C R D E D P  Rv3219
   4  E A P A P F E - E P L P P E A T D Q W Q D R A L C A Q T D P  Rv3260c
   3  Q P - - - - E Q L P G P N A D I W N W Q L Q G L C R G M D S  Rv3416


  19  E L W F P - - - D K G Q S - - - T R D - - A K R M C M R C P  WHIBTM4
  10  - - - - - - V G N S G A A - - - - A S - - A K M V C N R C -  CGLwhiB1
  13  E S F F P - - - E K G G S - - - T R E - - A K K V C L A C E  SCOELWB!
  43  D E L F V - - - - R G A A - - - Q R K - - A A V I C R H C P  MB3706c
  26  D L W F A - - - - D T P A - - - G L E V - A K T L C V S C P  MB3221c
  15  E L F F P - V G N S G P A L A Q I A D - - A K L V C N R C P  MB3245
  33  E A F F P - - - E K G G S - - - T R E - - A K K I C M G C E  MB3288c
  29  S M F F H P D G E R G R A R T - Q R E Q R A K E M C R R C P  MB3450
  43  D E L F V - - - - R G A A - - - Q R K - - A A V I C R H C P  ML2307c
  29  S M F F H P D G E R G R A R M - Q R E Q R A K E M C R R C P  ML0382c
  22  D L W F A - - - - E T P A - - - D L E C - T K T L C A N C P  ML0639
  33  E A F F P - - - E K G G S - - - T R E - - A K K I C L G C E  ML0760
  15  E L F F P - V G N S G P A I A Q I A D - - A K L V C N R C P  ML0804c
  15  E L F F P - V G N S G P A L A Q I A D - - A K L V C N R C P  MAPwhib1
  15  E L F F P - V G N S G P A L A Q I A D - - A K L V C N R C P  MAviumwhiB1
  19  D L W F A - - - - E A P A - - - D L E R - A K Q L C A G C P  MAvium290
  56  E A F F P - - - E K G G S - - - T R E - - A K K I C L G C E  MAvium364
  11  S M F F H P D G E R G R A R M - Q R E Q R A K E M C R Q C P  MAvium496
   1  - - - - M - - - - R G A A - - - Q R K - - A A V I C R H C P  MAvium729
  49  D E L F V - - - - R G A A - - - Q R K - - A A V I C R H C P  MS6199
  29  S M F F H P D G E R G R A R A - Q R E M R A K E M C R S C P  MS1597
  73  E A F F P - - - E K G G S - - - T R E - - A K R I C Q G C E  MS1831
  15  E L F F P - V G N S G P A L A Q I A D - - A K L V C N R C P  MS1919
 115  D L W F A - - - - E N P G - - - D L E R - A K A L C A G C P  MS1953
  43  D E L F V - - - - R G A A - - - Q R K - - A A V I C R H C P  Rv3681c
  68  D L W F A - - - - D T P A - - - G L E V - A K T L C V S C P  Rv3197A
  15  E L F F P - V G N S G P A L A Q I A D - - A K L V C N R C P  Rv3219
  33  E A F F P - - - E K G G S - - - T R E - - A K K I C M G C E  Rv3260c
  29  S M F F H P D G E R G R A R T - Q R E Q R A K E M C R R C P  Rv3416

  

    
  41  L L D E C R A L A L R D P H L V G V W G G - L S A Q E R - R  WHIBTM4
  27  V T S - C - A W A - - T G - D A G V W G G - M S D - - R - R  CGLwhiB1
  35  V R S E C L E Y A L A N D E R F G I W G G - L S E C E R      SCOELWB!
  64  V M Q E C A A D A L D N K V E F G V W G G - M T E R Q R R A  MB3706c
  48  I R R Q C L A A A L Q R A E P W G V W G G E I F D Q G S I V  MB3221c
  42  V T T E C L S W A L N T G Q D S G V W G G - M S E D E R - R  MB3245
  55  V R H E C L E Y A L A H D E R F G I W G G - L S E R E R - R  MB3288c
  58  V I E A C R S H A L E V G E P Y G V W G G - L S E S E R D L  MB3450
  64  V M Q E C R A D A L D N K V E F G V W G G - M T E R Q R R A  ML2307c
  58  V I E E C R A H A L D V G E P Y G V W G G - L S E S E R D L  ML0382c
  44  I R R P C L E A A M E R A E P W G V W G G E I F D R G L I V  ML0639
  55  V R H E C L E Y A L A H D E R F G I W G G - L S E R E R - R  ML0760
  42  V T T E C L A W A L N T G Q D S G V W G G - M S E D E R - R  ML0804c
  42  V T T E C L G W A L N T G Q D S G V W G G - M S E D E R - R  MAPwhib1
  42  V T T E C L G W A L N T G Q D S G V W G G - M S E D E R - R  MAviumwhiB1
  41  V R R Q C L A A A L E R A E P W G V W G G E I L D R G A V L  MAvium290
  78  V R H E C L E Y A L E H D E R F G I W G G - L S E R E R - R  MAvium364
  40  V I Q E C R S H A L E V G E P Y G V W G G - L S E S E R D L  MAvium496
  18  V M Q E C G A D A L D N R V E F G V W G G - M T E R Q R R A  MAvium729
  70  V I L E C G A D A L D N R V E F G V W G G - M T E R Q R R A  MS6199
  58  V I A Q C R S H A L A V G E P Y G I W G G - L S E S E R E L  MS1597
  95  V R D A C L E Y A L A H D E R F G I W G G - L S E R E R - R  MS1831
  42  V T T E C L S W A L E S G Q D A G V W G G - M S E D E R - R  MS1919
 137  I R V Q C L T A A L E R Q E P W G V W G G E I L D R G S I V  MS1953
  64  V M Q E C A A D A L D N K V E F G V W G G - M T E R Q R R A  Rv3681c
  90  I R R Q C L A A A L Q R A E P W G V W G G E I F D Q G S I V  Rv3197A
  42  V T T E C L S W A L N T G Q D S G V W G G - M S E D E R - R  Rv3219
  55  V R H E C L E Y A L A H D E R F G I W G G - L S E R E R - R  Rv3260c
  58  V I E A C R S H A L E V G E P Y G V W G G - L S E S E R D L  Rv3416


      
  69  R I R - - - - - - - - - - - - - - - K G A S - - - - - - - A  WHIBTM4
  48  A - K R R K - - - - - - - - - - - N R G R G R - - - - - - A  CGLwhiB1
  61                                                               SCOELWB!
  93  L L K Q H P E V V S W S D Y L E - K R K R R T G T - - - - A  MB3706c
  78  S H K R - - - - - - - - - - - - - P R G R P R K D - - - - A  MB3221c
  70  A L K R R N - - - - - - - - - - - A R T K A R - - - - - - T  MB3245
  83  R L K - - - - - - - - - - - - - - - R G I I .                MB3288c
  87  L L K G T - - - - - - - - - M G R T R G I R R - - - - - - T  MB3450
  93  L L K Q H P E V V S W A D F F D - T R K H R N - - - - - - V  ML2307c
  87  L L K G D - - - - - - - - - L A R S R S I P R - - - - - - S  ML0382c
  74  S R K R - - - - - - - - - - - - - P R G R P C N D - - - - V  ML0639
  83  R L K - - - - - - - - - - - - - - - R G V I .                ML0760
  70  A L K R R N - - - - - - - - - - - T R T K A R - - - - - - S  ML0804c
  70  A L K R R N - - - - - - - - - - - A R T K A R - - - - - - S  MAPwhib1
  70  A L K R R N - - - - - - - - - - - A R T K A R - - - - - - S  MAviumwhiB1
  71  G F K R - - - - - - - - - - - - - P R G R P R K D - G R R A  MAvium290
 106  R L K - - - - - - - - - - - - - - - R G I I                  MAvium364
  69  L L K G E - - - - - - - - - I G R G R G I R R - - - - - - S  MAvium496
  47  L L K Q H P E V V S W A D F F D - K R R N R S - - - - - - A  MAvium729
  99  L L K Q H P E V S S W A D F F A A Q R K H R S - - - - - - A  MS6199
  87  L L K - - - - - - - - - - - - - - - R G I R R - - - - - - S  MS1597
 123  R L K - - - - - - - - - - - - - - - R G I I .                MS1831
  70  A L K R R N - - - - - - - - - - - A R T K A R - - - - - - T  MS1919
 167  A R K R - - - - - - - - - - - - - P R G R P R K D S G G N P  MS1953
  93  L L K Q H P E V V S W S D Y L E - K R K R R T G T - - - - A  Rv3681c
 120  S H K R - - - - - - - - - - - - - P R G R P R K D - - - - A  Rv3197A
  70  A L K R R N - - - - - - - - - - - A R T K A R - - - - - - T  Rv3219
  83  R L K - - - - - - - - - - - - - - - R G I I .                Rv3260c
  87  L L K G T - - - - - - - - - M G R T R G I R R - - - - - - T  Rv3416

 

     
  77  .                                                            WHIBTM4
  60  R A V                                                        CGLwhiB1
  61                                                               SCOELWB!
 118  G .                                                          MB3706c
  91  V A .                                                        MB3221c
  83  G V .                                                        MB3245
  90                                                               MB3288c
 102  A .                                                          MB3450
 116  S .                                                          ML2307c
 102  A .                                                          ML0382c
  87  V V V .                                                      ML0639
  90                                                               ML0760
  83  G V .                                                        ML0804c
  83  G V                                                          MAPwhib1
  83  G V                                                          MAviumwhiB1
  87  G A A A                                                      MAvium290
 112                                                               MAvium364
  84  A                                                            MAvium496
  70  G                                                            MAvium729
 123  V .                                                          MS6199
  96  A .                                                          MS1597
 130                                                               MS1831
  83  G V .                                                        MS1919
 184  A A A .                                                      MS1953
 118  G .                                                          Rv3681c
 133  V A .                                                        Rv3197A
  83  G V .                                                        Rv3219
  90                                                               Rv3260c
 102  A .                                                          Rv3416

  
